# Clinical Outcomes of Left Bundle Branch Area Pacing Compared with Biventricular Pacing in Patients with Heart Failure Requiring Cardiac Resynchronization Therapy: Systematic Review and Meta-Analysis

**DOI:** 10.31083/j.rcm2411312

**Published:** 2023-11-09

**Authors:** Georgios Leventopoulos, Christoforos K. Travlos, Virginia Anagnostopoulou, Panagiotis Patrinos, Angeliki Papageorgiou, Angelos Perperis, Chris P. Gale, Konstantinos Α. Gatzoulis, Periklis Davlouros

**Affiliations:** ^1^Department of Cardiology, General University Hospital of Patras, 26504 Patras, Greece; ^2^Leeds Institute of Cardiovascular and Metabolic Medicine, University of Leeds, LS2 9JT Leeds, UK; ^3^Leeds Institute for Data Analytics, University of Leeds, LS2 9JT Leeds, UK; ^4^Department of Cardiology, Leeds Teaching Hospitals NHS Trust, LS1 3EX Leeds, UK; ^5^First Cardiology Department, National and Kapodistrian University of Athens, Hippokration General Hospital, 11527 Athens, Greece

**Keywords:** left bundle branch area pacing, meta-analysis, resynchronization

## Abstract

**Background:**

Biventricular pacing (BVP) is recommended for patients with heart failure (HF) who require cardiac resynchronization therapy (CRT). Left bundle branch area pacing (LBBAP) is a novel pacing strategy that appears to ensure better electrical and mechanical synchrony in these patients. Our aim was to systematically review and meta-analyze the existing evidence regarding the clinical outcomes of LBBAP-CRT compared with BVP-CRT.

**Methods:**

Medline, Embase, Cochrane Central Register of Controlled Trials and Web of Science databases were searched for studies comparing LBBAP-CRT with BVP-CRT. Outcomes were all-cause mortality, heart failure hospitalizations (HFH) and New York Heart Association (NYHA) class improvement. We included randomized controlled trials (RCTs) and observational studies with participants that had left ventricular ejection fraction (LVEF) ≤40% and (i) symptomatic HF or (ii) expected ventricular pacing >40%. Random and fixed effects models pairwise meta-analysis was conducted. Cochrane Risk of Bias 2 assessment tool (ROB 2.0) and the Newcastle–Ottawa scale (NOS) were used to assess the quality of the studies.

**Results:**

Eleven studies (10 observational studies and 1 RCT) with 3141 patients were included in the analysis. Compared with BVP-CRT, LBBAP-CRT was associated with lower risk of all-cause mortality (risk ratio (RR): 0.71, 95% CI: 0.57 to 0.87; *p* = 0.001), lower risk of HFH (RR: 0.59, 95% CI: 0.50 to 0.71; *p* < 0.00001) and more improvement in NYHA class (weighed mean difference (WMD): –0.36, 95% CI: –0.59 to –0.13; *p* < 0.00001) compared with patients who received BVP-CRT.

**Conclusions:**

Compared with BVP-CRT, receipt of LBBAP-CRT in patients with HF is associated with a lower risk of mortality, and HFH and greater improvement in NHYA class.

## 1. Introduction

Biventricular pacing (BVP) is recommended from the most recent European guidelines as the first-line pacing strategy in patients with heart failure (HF) that require cardiac resynchronization therapy (CRT) [1]. Many studies have shown its beneficial effects on morbidity and mortality in this population [2, 3]. However, 10% of patients cannot be treated by BVP due to having an unsuitable coronary sinus vein, while 30-40% are non-responders to BVP and experience no benefit from this treatment [4]. Conduction system pacing (CSP) has emerged as a solution to CRT downsides and is represented by His Bundle Pacing (HBP) and Left Bundle Branch Area Pacing (LBBAP). Current data demonstrates that HBP offers preservation or even restoration in intra or interventricular synchrony. Thus, it can be applied in HF patients, but it is technically challenging and related to high pacing thresholds [5].

LBBAP is a new pacing modality that can achieve narrow QRS and improve left ventricular function in patients with HF, by engaging the intrinsic conduction pathway of the heart [6]. According to existing evidence, LBBAP results in similar or even better improvement in the electromechanical synchrony compared with BVP [7] and is currently the globally prevailing method of CSP. Nevertheless, a study that systematically synthesizes and exclusively analyzes the effect of LBBAP compared with BVP in hard clinical outcomes is still lacking.

We conducted a systematic review and meta-analysis of observational and randomized controlled trials comparing the two pacing modalities to examine the effectiveness of LBBAP-CRT on all-cause mortality, heart failure hospitalizations (HFH) and New York Heart Association (NYHA) class improvement in HF patients who require CRT.

## 2. Methods

This systematic review and meta-analysis were performed in accordance with the Preferred Reporting Items for Systematic Reviews and Meta-Analyses (PRISMA) guidelines. The protocol of the present study was not registered. All data used and analyses performed in this systematic review and meta-analysis were based on previously published studies.

### 2.1 Search Strategy and Inclusion Criteria

We systematically searched Medline, Embase, Cochrane Central Register of Controlled Trials (via Ovid framework) and Web of Science databases from inception to February 8, 2023, for studies comparing LBBAP with BVP for CRT in patients with HF. Search terms were “left bundle branch pacing” AND “biventricular pacing”. Clinical studies were included if they met the following criteria: (1) randomized controlled trials (RCTs) or observational trials that compared a LBBAP group (LBBAP-CRT) with a BVP group (BVP-CRT) for CRT in patients with HF; (2) studies comparing all-cause mortality and/or HFH rates and/or NYHA class improvement between the two groups; (3) the participants of the studies should have (i) symptomatic HF with left ventricular ejection fraction (LVEF) ≤40% or (ii) LVEF ≤40% and expected rate of ventricular pacing >40%.

We excluded: case reports, editorials, letters, review articles, congress abstracts, animal studies, studies in individuals aged <18 years, and studies including <10 participants.

Studies in which the study arm was referred as CSP and included both patients that received HBP and LBBAP were excluded as data exclusively for LBBAP could not be extracted and our aim was a pure comparison of LBBAP-CRT vs BVP-CRT.

### 2.2 Outcomes

The primary outcome was all-cause mortality from baseline to longest follow-up as defined in each study. Secondary outcomes were HFH and NYHA class improvement.

### 2.3 Data Extraction

Articles were screened for inclusion by two independent investigators (CT and GL) who also extracted data on all-cause mortality, HFH rates and NYHA class improvement, using the same Excel spreadsheet. Data regarding study characteristics, number of participants, patient baseline characteristics, duration of follow-up, inclusion criteria and procedural success rate were also collected. For each continuous data type, the sample mean and standard deviation were extracted. If the results were reported as median and interquartile range, we converted them using the Wan’s *et al*. [8] method, into sample mean and standard deviation. Data for all outcomes of interest were extracted at the longest follow-up time point.

### 2.4 Quality Assessment and Statistical Analysis

The quality of included studies was assessed by using the Newcastle–Ottawa scale (NOS) for observational studies and the Cochrane Risk of Bias 2 assessment tool (ROB 2.0) for RCTs. Data were pooled for each outcome of interest (mean value, standard deviation and sample size for continuous variables and number of events and sample size for dichotomous variables), to compare the outcomes between LBBAP-CRT and BVP-CRT groups. Weighed mean difference (WMD) was the effect measure for continuous variables while dichotomous variables were reported as risk ratio (RR) and 95% confidence intervals (CIs) were used both for continuous and dichotomous outcomes. A fixed-effects (Mantel–Haenszel) meta-analysis was conducted if $I^{2}$ statistic was <50%. Otherwise, a random-effects (DerSimonian-Laird) model was used considering the substantial heterogeneity. All *p* values were two-sided, with *p* < 0.05 considered as significant. All statistical analyses were performed using RevMan 5.4 software (The Cochrane Collaboration, The Nordic Cochrane Centre, Copenhagen, Denmark).

### 2.5 Sensitivity Analysis

Sensitivity analysis was performed for all the outcomes to explore the consistency of the results, by removing one study at one time (“leave-one-out sensitivity analysis”).

## 3. Results

### 3.1 Studies Selection

**Fig. 1.**
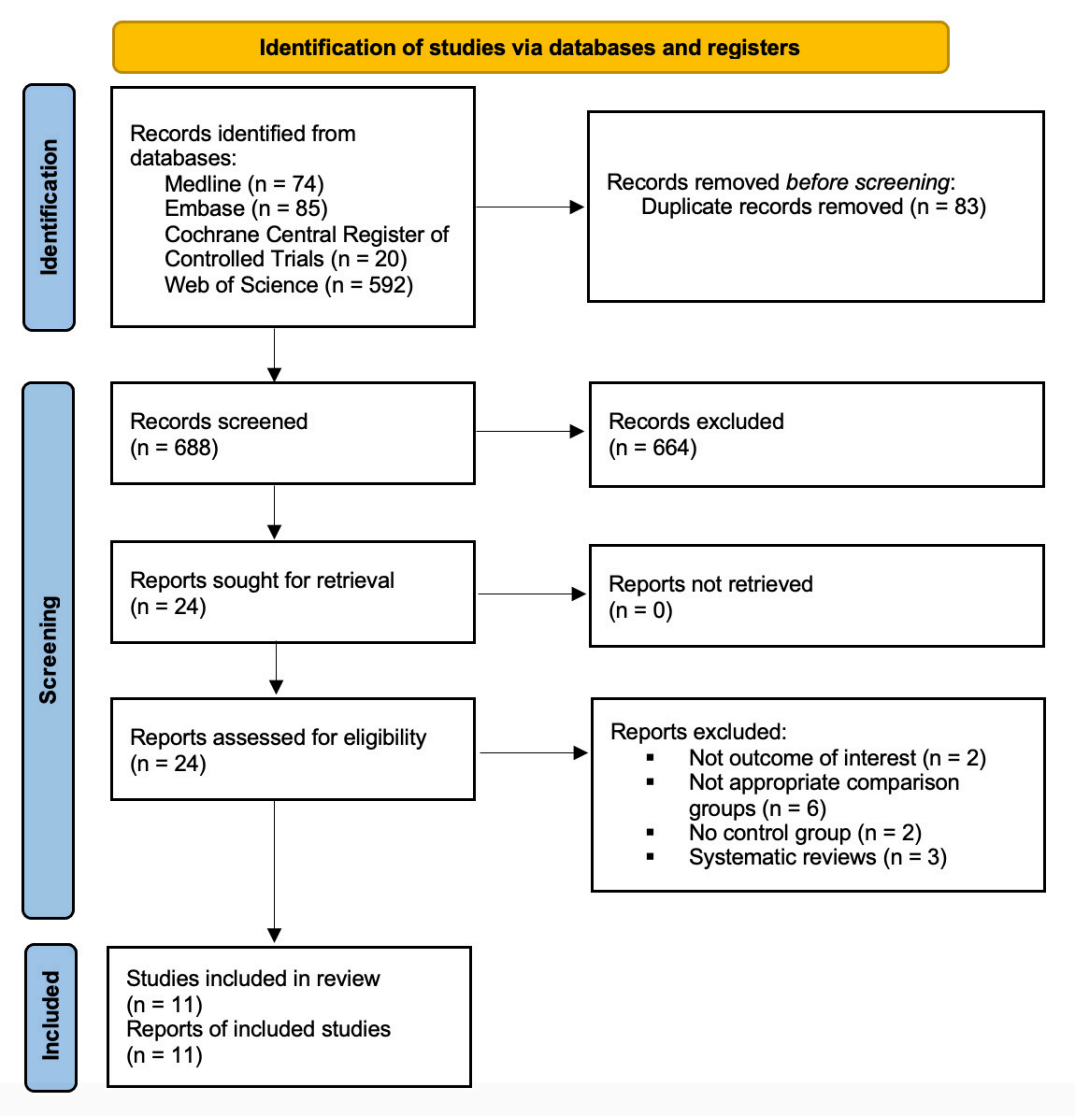
**Flow diagram of literature search**.

In total, 769 studies were retrieved and 11 were included in this systematic review and meta-analysis [7, 9, 10, 11, 12, 13, 14, 15, 16, 17, 18] (Fig. 1). Ten were observational studies [9, 10, 11, 12, 13, 14, 15, 16, 17, 18] and one was RCT [7]; all compared LBBAP-CRT with BVP-CRT providing data for outcomes of interest.

### 3.2 Characteristics of Included Studies

Patient baseline characteristics are presented in Table 1 (Ref. [7, 9, 10, 11, 12, 13, 14, 15, 16, 17, 18]). A total of 3141 individuals were enrolled in these 11 trials (1290 in the LBBAP-CRT group and 1851 in the BVP-CRT group). The mean follow-up duration was 14.6 ± 8.66 months and the average procedural success rate in the LBBAP-CRT group was 87.4%. The baseline characteristics were similar between the two groups. There were no significant differences regarding the mean age of the participants (66 ± 10, LBBAP-CRT vs 66 ± 10.1, BVP-CRT), the baseline LVEF (28.8 ± 6.1, LBBAP-CRT vs 28.9 ± 6.2, BVP-CRT) and the rate of patients diagnosed ischemic cardiomyopathy (ICM) (29%, LBBAP-CRT vs 30%, BVP-CRT). All observational studies were of good quality and the risk of bias in the RCT was low.

**Table 1.** **Patient baseline characteristics and details of included studies**.

Study	Centers (n)	Country	Study type	Treatment group	Patients (n)	Follow-up, months	Age, years	Male, %	Baseline LVEF, %	NICM (n)	ICM (n)	AF (n)	Inclusion criteria	Procedural success rate %	NOS scale and ROB 2.0
Chen *et al*. [9] 2022	4	China	Observational, prospective	LBBAP-CRT	49	12	67 ± 9	49	29.05 ± 5.09	36	13	4	HF, NYHA II-IV, LVEF ≤35%, LBBB	98	9
				BVP-CRT	51		64 ± 9	58	28.36 ± 5.30	41	10	3		NR	
Diaz *et al*. [18] 2023	5	International	Observational, prospective	LBBAP-CRT	128	11 ± 7	70 ± 10	69	25.20 ± 8.30	82	46	65	HF, NYHA II-IV, LVEF <35% + LBBB or LVEF <40% + VP >40%	84.4	9
				BVP-CRT	243		70 ± 12	71	26.70 ± 7.20	243	100	122		NR	
Guo *et al*. [10] 2020	1	China	Observational, prospective	LBBAP-CRT	21	14 ± 7	66 ± 10	43	30.00 ± 5.00	19	2	3	HF, NYHA II-IV, LVEF ≤35%, LBBB	87.5	9
				BVP-CRT	21		65 ± 8	43	29.80 ± 4.10	19	2	1		NR	
Hua *et al*. [11] 2022	1	China	Observational, prospective	LBBAP-CRT	21	24 ± 4	66 ± 7	71	30.05 ± 7.03	NR	NR	5	HF, NYHA II-IV, LBBB	NR	8
				BVP-CRT	20		68 ± 12	75	31.40 ± 9.30	NR	NR	4		NR	
Li *et al*. [12] 2020	3	China	Observational, prospective	LBBAP-CRT	27	6	58 ± 10	60	28.80 ± 4.50	23	4	5	HF, NYHA II-IV, LVEF ≤35%, LBBB	81.1	8
				BVP-CRT	54		59 ± 9	60	27.20 ± 4.90	46	8	11		NR	
Liang *et al*. [13] 2022	2	China	Observational, retrospective	LBBAP-CRT	154	31	67 ± 9	61	32.30 ± 6.70	126	28	46	HF, NYHA II-IV, LVEF ≤35%	94	9
				BVP-CRT	337		62 ± 10	70	30.30 ± 8.20	304	33	70		NR	
Rademakers *et al*. [14] 2023	1	Netherlands	Observational, prospective	LBBAP-CRT	31	6	68 ± 13	48	28.00 ± 8.00	20	11	9	HF, NYHA II-IV, LVEF ≤35%, LBBB	78	8
				BVP-CRT	40		71 ± 9	68	31.00 ± 6.00	26	14	13		NR	
Vijayaraman *et al*. [17] 2023	15	International	Observational, retrospective	LBBAP-CRT	797	33 ± 16	69 ± 12	64	27.00 ± 6.00	479	263	286	HF, NYHA II-IV LVEF <35% + indication for CRT or expected VP >40%	NR	9
				BVP-CRT	981		68 ± 12	70	26.00 ± 6.00	550	386	364		NR	
Wang *et al*. [15] 2020	1	China	Observational	LBBAP-CRT	10	6	65 ± 7	90	26.80 ± 3.85	9	1	NR	HF, NYHA II-IV, LVEF ≤35%, LBBB	100	7
				BVP-CRT	30		63 ± 10	77	26.38 ± 5.27	27	3	NR		NR	
Wang *et al*. [7] 2022	2	China	RCT	LBBAP-CRT	20	6	62 ± 11	35	28.30 ± 5.30	20	0	0	HF, NYHA II-IV, LVEF ≤40%, LBBB	90	Low
				BVP-CRT	20		65 ± 11	65	31.10 ± 5.60	20	0	0		NR	
Wu *et al*. [16] 2021	1	China	Observational, prospective	LBBAP-CRT	32	12	67 ± 13	44	30.90 ± 7.30	31	1	7	HF, NYHA II-IV, LVEF ≤40%, LBBB	NR	9
				BVP-CRT	54		68 ± 10	54	30.00 ± 6.20	47	7	11		NR	

LVEF, left ventricular ejection fraction; AF, atrial fibrillation; HF, heart failure; CRT, cardiac resynchronization therapy; NYHA, New York Heart Association; BVP, biventricular pacing; LBBAP, left bundle branch area pacing; ICM, ischemic cardiomyopathy; NICM, non ischemic cardiomyopathy; NOS, Newcastle-Ottawa scale; ROB 2.0, Cochrane Risk of Bias 2 assessment tool; LBBB, left bundle branch block; NR, not referred; VP, ventricular pacing.

### 3.3 Pairwise Meta-Analysis

**Fig. 2.**
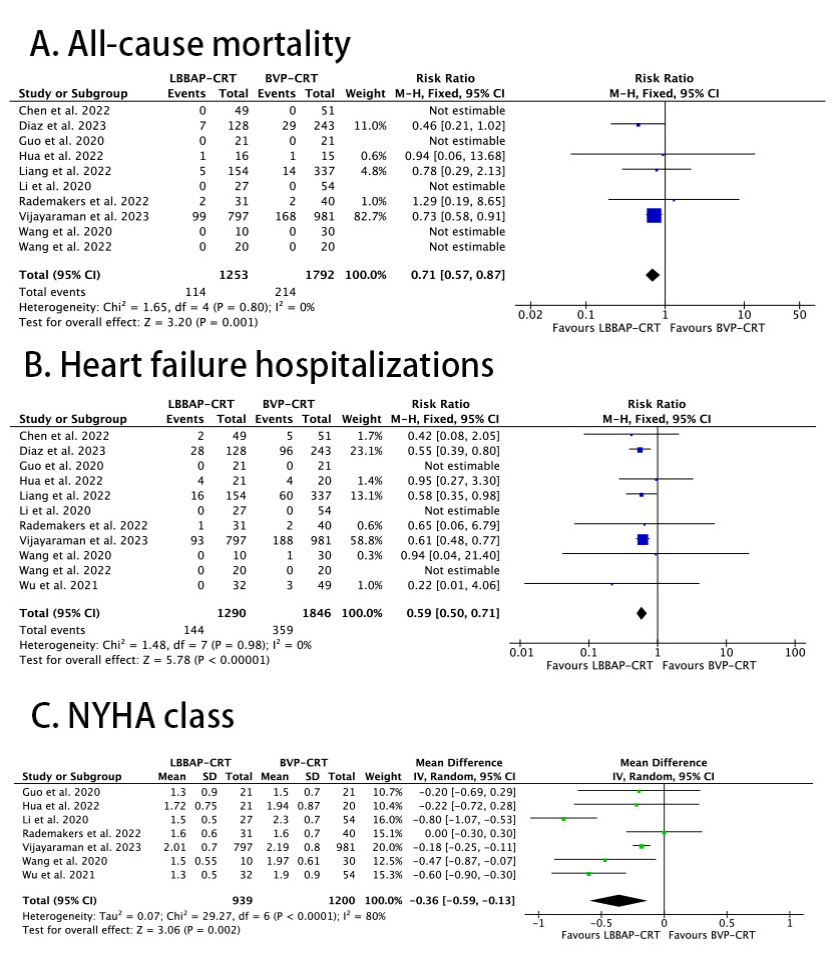
**Forest plots of LBBAP-CRT vs BVP-CRT for: (A) all-cause mortality; (B) heart failure hospitalizations; (C) NYHA class at longest follow-up**. CRT, cardiac resynchronization therapy; NYHA, New York Heart Association; BVP, biventricular pacing; LBBAP, left bundle branch area pacing.

The outcome of all-cause mortality was studied in ten trials including 3045 patients. In the LBBAP-CRT group, 114 events were reported (9%) vs 214 in the BVP-CRT group (11.9%). There was a statistically significant difference between the two groups (RR: 0.71, 95% CI: 0.57 to 0.87; $I^{2}$ = 0%; *p* = 0.001, Fig. 2A). The outcome of HFH was assessed in 11 studies including 3136 patients, with 144 events in the LBBAP-CRT group (11.1%) and 359 events in the BVP-CRT group (19.4%). LBBAP-CRT was associated with a lower risk of HFH compared with BVP-CRT (RR: 0.59, 95% CI: 0.50 to 0.71; $I^{2}$ = 0%; *p* < 0.00001) (Fig. 2B). The improvement of NYHA class from baseline to follow-up was assessed in seven studies including 2139 patients. Wang *et al*. [7] also provided data on NYHA class improvement but in a way different from all the other studies, so it was excluded from the analysis. NYHA class at baseline was similar in both groups and the analysis showed that at follow-up there was a statistically significant difference in favor of LBBAP-CRT (WMD: –0.36, 95% CI: – 0.59 to – 0.13; $I^{2}$ = 80%; *p* = 0.002) (Fig. 2C).

### 3.4 Sensitivity Analysis

Sensitivity analysis was performed to explore the consistency of the results, by removing one study at a time (“leave-one-out sensitivity analysis”). For the outcomes of HFH and NYHA class improvement the results remained robust. For the outcome of all-cause mortality, the result was found to be driven by Vijayaraman *et al*., 2023 [17]. Excluding this study from the analysis, there was no statistically significant difference between the two groups (RR: 0.61, 95% CI: 0.34 to 1.08; $I^{2}$ = 0%; *p* = 0.09).

## 4. Discussion

Reducing HFH symptoms and mortality is central to the management of patients with HF. Evidence from RCTs demonstrated that receipt of BVP-CRT for HF is effective in reducing mortality and HFH but does not allow the physiological activation of ventricles that LBBAP-CRT does. Also, the significant rate of non-responders to BVP remains an important drawback. A systematic review and meta-analysis has demonstrated the superiority of conduction system pacing—incorporating both HBP and LBBAP—compared to CRT in terms of electrical resynchronization, left ventricular ejection fraction, NYHA class improvement and rate of heart failure hospitalizations. All-cause death did not show any statistically significant difference between the two groups and the mean time of observation for this parameter was 11 ± 7.1 months [19].

A more updated systematic review and meta-analysis by Kim *et al*. [20] compared again CSP vs CRT in heart failure patients and the striking finding was a significant difference in all-cause mortality (odds ratio [OR] 0.68, 95% confidence interval [CI]: 0.56–0.83) with a median follow up time of 10.1 months. This discrepancy can be explained by the fact that more and larger observational studies were incorporated [17, 18].

Of note, both Vijayaraman *et al*. [17] (in multivariate analysis) and Diaz *et al*. [18] failed to demonstrate a clear benefit when analyzed separately on all-cause mortality.

Our aim was to focus strictly on LBBAP because it has now been adopted as the first-choice method in CSP by the majority of the operators and seems that this is the technique that will prevail over HBP in the future. In this systematic review and meta-analysis of 11 studies, we found that LBBAP-CRT is associated with lower mortality, lower risk of HFH than BVP-CRT and a greater improvement in NYHA class than BVP-CRT. However, it has to be highlighted, as stated above in the sensitivity analysis, that the outcome of all-cause mortality was mainly driven by Vijayaraman’s study [17]. Another older retrospective study by Vijayaraman including fewer centers, and as a result fewer patients, was conducted showing a smaller benefit of CSP over CRT in HFH and no difference on all-cause mortality. Moreover, the first chronological study had a follow-up of 27 ± 12 months [21], whereas the latest one [17] has a follow-up of 33 ± 16 months, which could explain the difference in results.

Vijayaraman *et al*. [17] in his more recent study reports a lower death rate (12% in CSP group vs 17% in BVP) compared to our meta-analysis (9% in LBBAP vs 11.9 in BVP) The most reasonable explanation for this difference is the longer follow-up period of Vijayaraman *et al*. [17] (33 ± 16 months) compared with our study (14.6 ± 8 months), as well as the older mean age of the participants (69 ± 12 years in Vijayaraman *et al*. [17] vs 66 ± 10 years in our study). Other factors that may contribute to the difference is the much higher percentage of patients with ICM in Vijayaraman *et al*. [17] (33% LBBAP–39% BVP) compared with (29% LBBAP–30% BVP) and it is well known that ICM is associated with less favorable outcomes in patients receiving BVP-CRT due to the overall scar burden [22]. Presence of scarring in LBBAP patients is a double-edged sword and the clinical outcome may be influenced by the location of fibrosis. If the scar is located laterally, LBBAP could be a preferable option as it removes the need of an left ventricular (LV) lead capture in a fibrotic area. On the other hand, a septal scar renders the advancement of the LBBAP lead difficult and increases the failure rate.

All-cause mortality benefit is the quintessence of a therapeutic intervention in medicine. This meta-analysis cannot provide robust data that could affect our daily clinical practice in terms of resynchronization in HF patients. It does though generate a strongly based hypothesis that should be further validated in a large, randomized study designed and powered to demonstrate all-cause mortality benefit, if this finally exists. Until then, the data presented above about all-cause mortality benefit should be interpreted with caution.

A number of observational studies [9, 10, 12] and one RCT [7] have found that LBBAP-CRT achieves better electromechanical synchrony in terms of QRS duration reduction and improvement of echocardiographic parameters compared with BVP-CRT, in the short-term follow-up. In BVP-CRT patients, electrical remodeling (native QRS shortening >10 msec post implant) seems to precede mechanical remodeling, and is an important factor for better clinical outcomes [23]. The effect of LBBAP on electrical remodeling should be further studied and could also explain its impact on HFH and NYHA class despite the quite short follow up in our meta-analysis.

This short-term predominance of LBBAP-CRT in the limited existing evidence seems to be translated into better clinical outcomes in terms of HFH rate and improvement in NYHA class. In the largest so far, a study comparing LBBAP-CRT to BVP-CRT in HF patients, Vijayaraman *et al*. [17], reports a HFH rate of 12% in LBBAP-CRT vs 19% in BVP-CRT [17]. These results are similar to our meta-analysis (11.1% in LBBAP-CRT vs 19.4% in BVP-CRT). Moreover, Vijayaraman *et al*. [17] proceeded to complete a sub-analysis in patients that had a left bundle branch block (LBBB) on their baseline electrocardiogram (ECG), whilst only 61% of the patients in his cohort had LBBB preimplant. The benefit of LBBAP is numerically larger if LBBB preexists. These better results of LBBAP-CRT in NYHA class and HFH in LBBB patients may be due to the fact that LBBAP can completely correct LBBB by placing the lead beyond the block site while BVP-CRT reduces the QRS without correcting the LBBB. This advantage may be the reason for the better electromechanical parameters of LBBAP-CRT that can lead to better clinical symptoms improvement. As in all-cause mortality, large multicenter, randomized controlled trials in different subgroups of patients (ICM – non-ICM) are needed to shed adequate light regarding benefit in HFH rate and NYHA class improvement.

### Limitations

Our study has certain limitations. First, ten out of 11 included studies were observational studies (with biases of confounding by indication and confounding), and the one RCT included was not sufficiently powered for the outcomes of interest. Thus, data from the RCT were pooled with that from observational studies which can lead to some uncontrolled bias. Second, the majority of the studies had a small sample size which can lead to inaccuracy of the effects. Third, most of the studies had a short follow-up period which is in contrast with the outcomes of interest that are considered as long-term. Fourth, some studies that explored patients with conduction system pacing including both LBBAP and HBP were excluded as data strictly about LBBAP could not be extracted. Fifth, patients that received both an LBBP lead and an LV lead as an optimized resynchronization strategy (left bundle branch optimised cardiac resynchonization treatment-LOT CRT) were excluded from our meta-analysis. Sixth, the protocol of this systematic review was not registered, and this fact may be considered as a limitation.

## 5. Conclusions

In our study, we showed that LBBAP-CRT has better results in all-cause mortality, HFH, and NYHA class improvement compared with BVP-CRT. However, larger, multicenter, randomized controlled trials are needed to verify our results concerning the clinical outcomes of this novel pacing method in patients with HF requiring CRT.

## Data Availability

Data are available upon reasonable request from the corresponding author at levent2669@hotmail.com.

## Supplementary Material

Supplementary material associated with this article can be found, in the online version, at https://doi.org/10.31083/j.rcm2411312.
